# Redefining the hypotheses driving Parkinson’s diseases research

**DOI:** 10.1038/s41531-022-00307-w

**Published:** 2022-04-19

**Authors:** Sophie L. Farrow, Antony A. Cooper, Justin M. O’Sullivan

**Affiliations:** 1 Liggins Institute, The University of Auckland, Auckland, New Zealand; 2 The Maurice Wilkins Centre, The University of Auckland, Auckland, New Zealand; 3 Australian Parkinson’s Mission, Garvan Institute of Medical Research, Sydney, New South Wales Australia; 4 School of Clinical Medicine, UNSW Sydney, Sydney, New South Wales Australia; 5 MRC Lifecourse Epidemiology Unit, University of Southampton, Southampton, United Kingdom

**Keywords:** Genomics, Parkinson's disease, Translational research, Systems biology

## Abstract

Parkinson’s disease (PD) research has largely focused on the disease as a single entity centred on the development of neuronal pathology within the central nervous system. However, there is growing recognition that PD is not a single entity but instead reflects multiple diseases, in which different combinations of environmental, genetic and potential comorbid factors interact to direct individual disease trajectories. Moreover, an increasing body of recent research implicates peripheral tissues and non-neuronal cell types in the development of PD. These observations are consistent with the hypothesis that the initial causative changes for PD development need not occur in the central nervous system. Here, we discuss how the use of neuronal pathology as a shared, qualitative phenotype minimises insights into the possibility of multiple origins and aetiologies of PD. Furthermore, we discuss how considering PD as a single entity potentially impairs our understanding of the causative molecular mechanisms, approaches for patient stratification, identification of biomarkers, and the development of therapeutic approaches to PD. The clear consequence of there being distinct diseases that collectively form PD, is that there is no single biomarker or treatment for PD development or progression. We propose that diagnosis should shift away from the clinical definitions, towards biologically defined diseases that collectively form PD, to enable informative patient stratification. N-of-one type, clinical designs offer an unbiased, and agnostic approach to re-defining PD in terms of a group of many individual diseases.

## Introduction

There is growing recognition that Parkinson’s disease is not a single entity^1,2^. Rather there are multiple different clinical, genetic and epidemiologically heterogeneous diseases that together are recognised within the one umbrella term of Parkinson’s disease^3–5^. Hereafter we refer to the multiple diseases as ‘**PD**’ for simplicity, and to prevent clouding the literature with a new term. Despite growing recognition of this concept, the majority of PD targeted research focuses on the ‘common’-pathological end-point of a linear PD storyline^6^: the physical manifestation of neuronal inclusions termed Lewy bodies, and the loss of dopaminergic neurons (DAn) within the central nervous system (CNS). This focus on the end-point pathology has proven its worth in the development of effective symptomatic therapies that include Levodopa^7^. However, the failure of nineteen phase 3 intervention trials^8^ targeting modification of disease progression illustrates a limitation of this focus. The restricted focus on endpoint pathology largely arises from issues including that PD diagnosis typically occurs many years after disease onset, predominantly on the basis of motor symptoms, and yet one can only study PD patients *after* this clinical diagnosis is made. The successful development of disease-modifying therapeutics has been further hindered by the absence of biomarkers, and more critically—the absence of informative, molecular mechanisms that define each of the individual disease***s*** that collectively form PD. This is reflected in a lack of PD intervention trials that target specific mechanistic changes in groups of individual patients defined according to the mechanism(s) that contribute to disease development/progression. The SURE-PD3 trial is an exception that targeted only individuals with low serum urate concentrations^9^. However, beyond the SURE-PD3 trial, there is typically no specific measurable biological signal for the success of a disease-modifying intervention for each disease within the PD umbrella^10^. Instead, we remain reliant on relatively insensitive and variable clinical measures of PD progression^8^.

The advent of genome-wide association studies (GWAS) has enabled the identification of variants associated with risk of disease development^11^, different rates of cognitive decline^12^, and different rates of progression for PD^13,14^. However, the conglomeration of datasets needed to achieve the sample size and statistical power required for GWAS perpetuates the one-disease model of PD, and overlooks the presence of multiple different clinical, genetic and epidemiologically heterogeneous diseases. In these situations, the conglomeration of data across multiple different Parkinson diseases dilutes the frequency of specific disease-associated variants and thus reduces the ability to identify those variants that contribute to the trajectory of each individual disease (i.e., the aetiology). As such, the integration of genetic and standardised clinical data into a coherent coordinated approach to slow or prevent PD development, is yet to materialise. Achieving this requires that we move away from a dependence on the shared terminal pathology and clinical definitions and develop a means for patient stratification, using specific genetic information, that is based upon a sound understanding of the aetiology of each contributing molecular disease. But how can you achieve this, when to study the different diseases you must first define them? Here we will discuss how this circular argument can be broken using genetic, molecular and clinical information to identify the different trajectories within PD, from a prospective, disease risk-driven perspective, that stratifies patients and therapeutics without a priori assumptions.

## Multiple disease trajectories beneath the Parkinson’s disease umbrella

In 2008, William Weiner wrote “there is no Parkinson disease”^1^ and suggested *Parkinson disease****s*** as a more fitting term for the observed multiple aetiologies. The term Parkinson diseases is consistent with the fact that there is no obvious, predictable disease trajectory following diagnosis, even in monogenic forms of the disease. Rather, each individual’s pathway is unique, or at most shared with a limited number of fellow patients^15^.

**Fig. 1:**
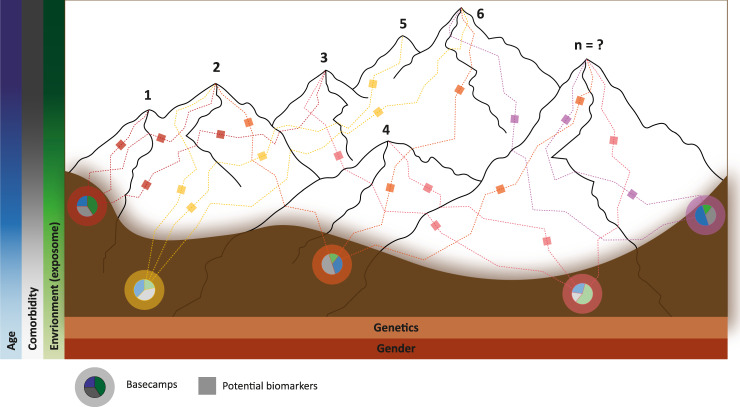
Parkinson Diseases Mountain Range model. Conceptual model assimilating the different diseases within PD to mountains within a range. There are likely many more mountains (diseases) than presented in this conceptual model. The topology of the valley floor represents the total variation in interaction between age, environment, comorbidities, sex and genetics of the population. An individual’s genetic risk is represented by the position in the valley (i.e., basecamp) where the individual starts climbing. Different signals (environment, age, comorbidity) from the dynamic basecamp surroundings interact with the individual’s genetic factors to alter aspects of the disease including onset age at which the patient begins climbing, or whether the individual even develops PD (reflected in the pie charts at base camps). The topology of the mountain (e.g. intrinsic and extrinsic factors) affects how quickly each individual climbs the route (i.e., the rate of disease progression), and thus the range, presentation and severity of symptoms^15^. The small boxes (i.e., checkpoints) along the routes of ascent represent potential biomarkers that could be developed/used to provide an unbiased snap-shot that can be used to track disease development within individual patients. However, these ‘on route’ biomarkers will likely change over the course of the disease.

To illustrate the impact that treating PD as a group of diseases with different but overlapping aetiologies^4,5,16^ can have on our understanding of the disease, let us consider a conceptual model where each disease within PD is represented by a mountain within a range of mountains (Fig. 1). At present we are unable to accurately define the number of different diseases that collectively form PD, thus we have limited our model to seven mountains, for simplicity. In the PD mountain range model, an individual’s genetic risk is represented by the position in the valley (i.e., basecamp) where the individual starts climbing—this position naturally limits the mountain(s) that can be ascended and the route(s) that can be taken. PD patients cluster according to their basecamp, of which there are a limited number, defined by the potential and realised combinations of the risk variants within the genome. Environmental signals from the dynamic basecamp surroundings interact with the individual’s genetic factors to alter aspects of the disease, including onset age at which the patient begins climbing, or whether the individual even develops PD. These environmental signals include, among others: pesticides and pollutants^17,18^, diet^19^, viral infection^20^, head trauma^21^, inflammatory diseases^22^ (for an in-depth review on the role of environmental signals in relation to PD genetics see Johnson et al.^23^). Once an individual has begun ascending a mountain, the topology of the mountain, which represents the intrinsic (e.g. the gut microbiome^24^ or comorbid disease pathology^25^) and extrinsic (e.g. exercise^26^, diet^19^, and periodic fasting^27^) factors, influences how quickly each individual climbs the route (i.e., the rate of disease progression), and thus the presentation and severity of symptoms^15^.

Individual diseases that together comprise PD are heterogeneous in and of themselves. This is represented in our model by the existence of multiple routes to each mountain summit. These routes are not independent, merging and diverging, meaning it is likely that individuals can switch between the routes dependent on their particular combination of intrinsic and extrinsic factors. Although heterogeneity likely exists within each disease, it would ideally be sufficiently homogeneous to provide a single therapeutic target for treatment development. Furthermore, each route has different markers, or checkpoints, at different stages—akin to the biomarkers that provide an unbiased snap-shot that can be used to track disease development within individual patients. It is important to note however that these ‘on route’ biomarkers will change over the course of the disease, and are likely to be influenced by the individual’s age, diet, and combination of predisposing comorbid diseases.

It can be argued that there are commonalities across individual diseases that contribute to PD (i.e., shared between the different mountains within the range). Treating these commonalities would provide treatment for a larger group of patients. This may be true. However, whilst potentially useful, treating these commonalities would have limited benefit, as the symptoms (e.g. resting tremor and bradykinesia) appear late in the disease course, and thus patients would be more disabled (closer to their respective summits) by the time the treatment is initiated. Notably, disease-modifying interventions that target PD based on this premise have yielded little success thus far.

Other models of PD have been presented before. Perhaps best known is William Langston’s elephant model^28^ which captures the idea of diverse symptomology but still presents PD as a single disease, or, elephant. In our model, the elephant would be represented as a single mountain within the PD mountain range. Thus Langston’s model does not capture the multiple diseases that collectively form PD, or the heterogeneity that is inherent to each disease.

## Using ‘omics to inform origins and trajectories of Parkinson’s disease

It is the patient’s combination of genetic risk coding (e.g. *LRRK2*-G2019S or *SNCA*-A53T) and non-coding variants that initially “set the stage” and determine which basecamp and mountain an individual will start ascending in their journey towards PD. The application of GWAS to the study of PD enables unbiased population-level identification of the genetic basis of risk that exist long before the disease initiates. However, the genetic variants that have been associated with PD by GWAS (e.g. 90 genetic loci^11^) only explain between 16-36% of the heritability of PD. Additionally, apart from a few exceptions, the odds ratios of the individual variants are typically low (e.g. between 0.8 – 1.2)^11^. Indeed, the current predictive ability of the SNPs associated with PD is so low as to make meaningful risk score prognosis unfeasible^29^. The missing heritability can partly be explained by issues with merging the multiple different diseases that contribute to PD, into the single entity that is defined by late-stage pathological markers (i.e., performing GWAS from the perspective of PD being a single disease). Furthermore, the reliance on a clinical definition means that no two ‘omics studies yield similar results since they only represent those of the heterogeneous patient population from which they were applied (e.g.^30^). Averaging these different but related datasets results in the identification of only the most significant risk loci that are common across all the diseases reaching statistical significance. The issue of averaging signals across the heterogeneous diseases that contribute to PD, when undertaking a GWAS, can be addressed by stratifying PD patients according to their genetically defined start-point, in turn enabling selection of informative longitudinal biomarkers and effective therapeutic approaches (specific to each route). This stratification can be achieved through genomic approaches that explore the specificities of GWAS manifestation^31,32^, and inform the distinct routes of PD development. As such, GWAS-based patient stratification could indicate 1) which pathway(s) is dysregulated; 2) pathway biomarkers to be examined; and 3) which targets should be considered for therapeutic intervention. However, shifting from simply identifying GWAS signals to informative stratification requires in depth characterisation of the causative variant(s) function^33^.

Until recently^33^, our inability to functionally translate non-coding genetic variation and risk to biologically disease-relevant pathways has meant that the earlier stages of PD development have been primarily neglected as a means of stratification or therapeutic intervention. In contrast to the noncoding risk variants, coding mutations in *GBA* and *LRRK2* genes have been explored and enabled patients with these specific mutations to be stratified for therapeutic intervention, targeting these genetic subgroups of patients^34,35^. Furthermore, Szwedo et al. demonstrated a role for *APOE-ε4* and *GBA* mutations in the rate of cognitive decline in PD patients, but found no significant impact for common variants in *SNCA* and *MAPT*^12^. These findings raise the possibility for earlier identification and stratification of individuals at high risk of rapid cognitive decline, thus highlighting suitable candidates for future targeted trials. Despite progress, the known incomplete penetrance of these mutations is problematic^36^ and highlights a remaining knowledge gap surrounding the mechanistic role of some of these mutations, such as the role of *LRRK2* mutations in disease progression^14^. This therefore raises the question as to whether such interventions will be effective against disease progression even in patients with these specific mutations. Nonetheless, with recent advancements, our understanding of how both coding and non-coding risk manifests is evolving^33,37^. Such understanding can be used to inform hypotheses which will aid in the identification and stricter classification of individual diseases within PD that could also lead to targeted therapeutics.

Functional characterisation requires that the associated molecular, cellular and physiological phenotyping is sufficiently deep to allow accurate assignment of the causal variants and their target genes^38^, and potentially what tissue(s), the disease risk is conveyed in. Panyard et al. applied an approach to functionally characterise and assign the action of causal genetic variants in Alzheimer’s disease (AD)^39^. Briefly, Panyard et al. integrated genomic and clinical data from two longitudinal AD cohorts with epigenetic annotations to develop cell-type-specific genomic functional annotations^39^. These annotations were used to identify which SNPs are likely to be functional in different tissues^39^. The authors demonstrated that effects of these SNPs in the liver were statistically associated with Alzheimer’s diagnosis^39^. In so doing, Panyard et al. highlighted a potential contribution from the liver towards AD, including associations with core AD cerebrospinal-fluid biomarkers, in what is widely considered a ‘brain-centric’ disease. Whilst a small study (*n* = 79 AD patients), the finding that changes in the liver were predictive for some, but not all, individuals is consistent with the hypothesis that the liver malfunction accounts for one of the heterogeneous diseases that collectively contribute to AD^40^.

Genomic approaches are also being applied in attempts to identify and understand the cell- and tissue- types where genetic risk manifests in PD^41,42^. For example, Coetzee et al.^43^ used histone modification data combined with enrichment analyses to demonstrate that many PD-associated genetic variants were enriched, and had expression quantitative trait loci (eQTL) associations, in non-neuronal cell-types, including lymphocytes, mesendoderm-, liver- and adipocyte- cells^43^. Similarly, we have used a discovery-based approach to identify putative regulatory impacts of non-coding PD-associated risk variants in both the CNS and peripheral tissues^33,44^. Notably, our analyses indicated that eQTL effects for a subset (28%) of the 90 PD-associated risk variants were only detected in peripheral tissues (e.g. thyroid and oesophagus)^33^ while only 2% of PD risk SNPs had identifiable eQTLs solely in CNS tissues. Given that tissues are complex mixtures of cell types, the oesophageal finding does not imply that the effect is due to the muscles at the exclusion of the nerves that innervate the oesophagus. However, the finding is consistent with peripheral symptomology (e.g. dysphagia), that is sometimes observed in the early stages of PD^45^.

In an attempt to determine which tissues, and subsequently cell-types, are responsible for PD heritability, Reynolds et al.^41^ used stratified Linkage Disequilibrium score regression^46^ (see box 1) to measure the contribution(s) that common genetic variation makes to the heritability of PD across 53 tissues (inc. 13 brain region tissues), using schizophrenia as a comparative measure. In contrast to schizophrenia in which all 13 brain tissues were significantly enriched for heritability, there was no enrichment for PD heritability across any of the 53 tissues (in the CNS or peripheral tissues). The lack of PD heritability enrichment across these bulk tissues led Reynolds et al. to question whether cellular heterogeneity within tissues may be masking signals, and thus sought to investigate cell-type-specific enrichment of heritability. However, across 6 human and 30 mouse CNS cell-types, Reynolds et al. identified no cell-type enrichment for PD heritability. The Lewy Body pathology in specific neuronal cell types, associated with PD, has encouraged researchers to focus efforts towards understanding risk in neuronal subtypes. However, the findings from Reynolds et al. provide reason to believe that risk loci are affecting non-CNS cell-types and/or cellular processes and pathways across multiple cell types, and to which different cell types have varying vulnerability^41^. Such varying vulnerability, consistent with the proposed threshold theory for PD^47^, could likely be a result of interactions with environmental factors and/or comorbid disease pathology.

In contrast to the lack of cell-type heritability enrichment identified by Reynolds et al., there have been multiple studies to date that implicate glial cell types, mostly microglia, in neuroinflammation and PD pathogenesis^42,48,49^. Given these implications, Bryois et al. combined cell-type-specific gene expression and GWAS data to explore the role of glial cells in PD pathogenesis^49^. Roles for microglia were indicated by the finding that cell-type-specific ATACseq identified functional PD risk loci that were enriched for autophagy and lysosomal processes^50^, both of which have been previously implicated in PD^51^. Furthermore, elevated *LRRK2* expression, associated with the linked PD GWAS SNPs rs76904798 and rs7294619 (*R*^2^ = 0.842), has also been shown to occur specifically in microglia^42^. Collectively, these data are consistent with the hypothesis that PD genetic risk variants affect non-neuronal cell types of the CNS. However, while these studies highlight the importance of cell-type consideration, they are still driven by a priori assumptions that are CNS focused. As such, it is essential to extend these analyses to non-CNS cell-types, following a more discovery-based, hypothesis-free approach, to determine if such risk enrichment is truly specific to the microglia, or if other non-CNS cell-types may also be involved in disease initiation and propagation.

Together these studies highlight how multiple ‘omics approaches can be used to identify the tissue- and cell-type-specific manifestations for GWAS risk variants. The findings we have discussed support two potential, non-mutually exclusive, hypotheses: First, the individual diseases within the PD umbrella may arise through genetic variation-dependent mechanisms that dictate the tissue-of-origin(s) and thus the pathological pathways associated with the disease. This concept is reflected in the mountain range model, with each basecamp representing a different, genetically-informed, start-point. In the second hypothesis, variants impacting a specific peripheral tissue- or cell-type, cause dysregulation that adds to the disease complexity/symptoms without necessarily leading to the CNS pathology that is typically associated with PD. This second hypothesis aligns with the threshold theory for PD which was developed on the basis of parallel degeneration of both the central and peripheral nervous systems^47^. As such, there is a need to look beyond the tissue- and cell-types that are traditionally associated with PD pathology to gain a greater understanding of the mechanisms through which genetic risk may be manifested. Advances within the fields of single-cell transcriptomics^52,53^ and bulk-cell analyses^54^ will provide additional insights that begin to untangle the relative contributions of genetics and the environment to PD risk manifestation. But the question remains, how do we apply these approaches to a mechanistically-heterogeneous disease?

Box 1 Glossary of terms. **Expression quantitative trait loci (eQTL):** A genetic locus that affects (or correlates with) the expression (mRNA) of one or more genes.

**Genome wide association study (GWAS):** An approach used to associate specific genetic variations with particular diseases or traits. The genomes of individuals with the disease or trait of interest are compared to the genomes of matched, control, individuals – to identify variants that are significantly associated with that particular disease or phenotypic trait.

**Infinitesimal model:** A model built on the premise that the inheritance of a quantitative trait is controlled by an infinite number of loci, and each locus has an infinitely small effect.

**Linkage disequilibrium (LD):** The non-random association of alleles at different loci.

**Linkage disequilibrium score regression (LDSC):** A statistical method for quantifying the separate contributions of polygenic effects and various confounding effects, such as population stratification, based on summary statistics from GWA studies.

**N-of-1 approach:** In this context, an n-of-1 analysis is a meta-analyses of deeply characterised single patient information, of individuals within a heterogeneous cohort, that explores genetic variation in the context of the measured phenotype(s). In effect, the characteristics of each participant are individually (and frequently where possible) noted and contrasted to each other individual. This approach accounts for the individual-level heterogeneity that is present in PD.

**Omnigenic model of complex disease:** The model is centred on the premise that human genome regulatory networks are hugely interconnected, and almost any gene with regulatory variants in at least one relevant tissue will contribute to the heritability of the phenotype.

**Single nucleotide polymorphism (SNP):** The most common type of variation among people. One SNP represents a variation at a single position (i.e. nucleotide) in the DNA sequence.

## Using big data to identify individual trajectories in a heterogeneous disease

Conglomerating data from different cohorts provides a large sample size *(n)* which is otherwise unachievable from a single-centre cohort. As such, conglomerated data provides much-needed statistical power to address particular hypotheses. Despite providing statistical power, the conglomeration of different PD cohorts unfortunately also highlights the lack of strict diagnostic criteria for PD and related diseases, with different cohorts often using different diagnostic criteria^30^. A further confounding problem, that affects diagnosis even at the level of a single clinician, is misclassification^55^. Such misclassification raises the problem of inclusion of non-PD patients in cohorts, which may be skewing outcomes of observational studies and clinical trials. A third, and substantial, complicating factor is the likely multiple different mechanistic diseases that exist within the ‘homogenous’ clinical PD cohorts currently studied. This problem is particularly prevalent in cohorts that include patients with different genetic predispositions to diseases within PD, such as *GBA-*PD and *LRRK2-*PD patients, who typically present with different symptomatic trajectories^56,57^. Grouping these different individual diseases together is likely causing a loss of information. If data conglomeration is to achieve what is hoped, disease biomarkers, and more specifically biomarkers for the different diseases that collectively form PD are urgently needed. The need to define individual diseases as opposed to merging them into a single entity is in line with the prediction made by Espay and Lang that smaller, smarter clinical trials are needed to move away from this ‘homogeneous’ clinical Parkinson’s phenotype^6^.

As discussed earlier, genetic risk variants offer an option for such genetic stratification—with an individual’s risk profile determining their disease starting point (e.g. specific basecamp in the mountain range model). These genetic risk variants, or SNPs, do not however act independently^33^. Rather they act in a combinatorial manner within a much larger genetic background. In order to understand the full contribution that PD-associated SNPs make to PD, they need to be considered in the context of the omnigenic^58^ and infinitesimal^59^ models for disease (see box 1), and in terms of network medicine^60^. Network medicine approaches enable the disease to be contextualised as a sum of inter-connected perturbations, reflective of the underlying genetic and molecular risk drivers (i.e. studying PD risk variants in the context of an individual’s complete genotype). The utility of network medicine^60^ has being explored in other complex diseases, and has already aided in the identification of novel targets for therapeutic strategies and development^61,62^.

Exploring the impact and interconnectivity of genetic contributions to an individual’s disease risk profile, from a network medicine angle, has only become feasible following recent advancements. These include the reductions in costs for genome sequencing and computing^63^, and the development of machine learning approaches to detect complex patterns in genomes. Such advances have informed, and been enhanced by, the rapidly evolving post-GWAS genome-editing toolbox, including CRISPR screens^64^ and massively parallel reporter assays^65^ (to test observed patterns for functional significance). These tools will over time provide the data required to understand the complete genetic contributions to the development of the diseases that collectively form PD, amongst other complex diseases. Collaborative efforts, such as the Atlas of Variant Effects Alliance (https://www.varianteffect.org/)^66^, will be critical in enabling the curation and systematic collation of results from these functional post-GWAS studies. Another recent technological advance that will likely enhance genomic findings from a phenotypic perspective is the introduction of wearables^67^. Such devices have been shown to provide vital sign data (e.g. heart rate and electrodermal activity) at a level equivalent to that gained in a clinical setting^68^. The widespread uptake of these wearables enables individualised, longitudinal and continuous health monitoring. While identifying signal from noise in movement measurements is challenging, combining the in-depth phenotypic data that wearables provide with matched genetic data promises to aid in identifying clinical differences amongst the different genomic diseases within PD.

Information on genetic variation and drug responses can be used to help determine which drugs, are likely to be safe and efficacious in an individual. These approaches are leading to the emergence of ‘genetically-informed’ clinical trials (i.e., precision medicine approaches) in PD^69–71^. For example, *Ambroxol* has been repurposed to treat PD patients with a *GBA* coding mutation^34^. Despite having only been trialled in a small, open label, non-randomised group of individuals, *Ambroxol* shows promise for the treatment of this well-defined yet heterogeneous (i.e., it included multiple *GBA* coding mutations) subset of individuals^34^. The *Ambroxol* trial is an exemplar that paves the way for future precision-informed clinical trials in PD. Not only does it address the issue of treating patients according to genomic information, but also shows the potential of repurposing already licensed medication^72^, to accelerate the process of drug development. The *Ambroxol* trial also included some idiopathic PD patients—of whom also showed promising responses to the treatment. Identifying idiopathic PD patients who specifically have reduced *GCase* activity (i.e., those with *GBA* modifying genotypes^30,44^) may lead to better outcomes for patients.

Despite the obvious promise of a stratified approach to clinical testing and therapy, the lack of genotyping as a part of clinical assessment means that the identification of the relatively small numbers of individuals with genetic predispositions remains a major financial and temporal challenge. However, this is changing as initiatives, such as PD frontline (https://pdfrontline.com/en) and PD GENEration (https://www.parkinson.org/PDGENEration), are offering genetic testing for PD patients to ensure individuals carrying defined mutations are referred to the clinical trials best suited to them.

## Concluding remarks & future perspectives

Recognising that many diseases contribute to PD highlights a challenge that is present in the search for a biomarker of PD progression and therapeutics. Specifically, if there are many diseases subsumed within the umbrella of PD, then we should be looking for biomarkers for each individual disease. That we continue selecting patients on the basis of clinical criteria rather than biological ones impairs our ability to do this. Even genetic risk for PD is currently viewed within the context of the shared pathology that connects the different Parkinson disease**s**. The utility of network medicine^60^ has been established in other complex diseases, aiding the identification of novel targets for therapeutic strategies and development^61,62^. While it is certainly true that further initiatives involving large-scale data conglomeration will aid in the molecular and clinical understanding of the disease, the lack of uniformity in PD diagnosis and disease trajectories will likely confound findings from genomic and biomarker studies^30,73^. Recent initiatives (e.g. PREDICT-PD^74^ and the Cincinnati Cohort Biomarker Program (CCBP)^75^) that incorporate discovery-based analyses of prospective cohorts are seeking to address this by defining PD developmental pathways and biomarkers. Furthermore, we contend that it is time to consider systematic n-of-1^76–78^ approaches (see box 1) in PD research, to identify the combinations and relative contributions of the genetic, pathological and environmental factors in each unique circumstance^3,79^, for individuals within a heterogeneous population. The population’s use of wearables will contribute to the collection of relevant data for achieving such an approach^80^. Ultimately, the aggregated results of n-of-1 approaches will help elucidate the many diseases that contribute to the one complex Parkinson disease. Redefining the hypotheses driving PD research will enable movement away from the current focus on shared pathology and clinical definitions. This in turn will make way for the development of targeted diagnostic and therapeutic approaches that are based upon a molecular understanding of the aetiology of the individual diseases, and thus have the ability to slow, stop or reverse disease progression and ultimately achieve disease prevention.

### Reporting Summary

Further information on research design is available in the Nature Research Reporting Summary linked to this article.

## Supplementary information

npj Checklist

Reporting Summary Checklist

## Data Availability

There is no data to share that is specific to this perspective paper. All information is included in the references.
